# Access to Medicines by Seguro Popular Beneficiaries: Pending Tasks towards Universal Health Coverage

**DOI:** 10.1371/journal.pone.0136823

**Published:** 2015-09-25

**Authors:** Edson Servan-Mori, Ileana Heredia-Pi, Julio Montañez-Hernandez, Leticia Avila-Burgos, Veronika J. Wirtz

**Affiliations:** 1 Center for Health Systems Research, National Institute of Public Health, Cuernavaca, Morelos, Mexico; 2 Department for Global Health, Boston University School of Public Health, Boston, United States of America; Kenya Medical Research Institute - Wellcome Trust Research Programme, KENYA

## Abstract

**Objective:**

In the context of aiming to achieve universal health coverage in Mexico, this study compares access to prescribed medicines (ATPM) between Seguro Popular (SP) and non-SP affiliated outpatient health service users.

**Materials and Methods:**

ATPM by 6,123 users of outpatient services was analyzed using the National Health and Nutrition Survey 2012. Adjusted bi-probit models were performed incorporating instrumental variables.

**Results:**

17.3% of SP and 10.1% of the non-SP population lacked ATPM. Two-thirds of all outpatient SP and 18.5% of all outpatient non-SP received health services at Ministry of Health facilities, among whom, 64.6 and 53.6% of the SP and non-SP population respectively reported ATPM at these facilities. Lack of medicines in health units, chronic health problems (compared to acute conditions) and prescription ≥3 medicines were risk factors for non-ATPM. Adjusted models suggest that when using Ministry of Health services, the SP population has a higher probability of ATMP compared to the non-SP population.

**Conclusion:**

Given the aspirations of achieving universal health coverage in Mexico, it is important to increase ATPM in Ministry of Health facilities thereby ensuring basic rights to health care are met.

## Introduction

Medicines are an essential element of a functioning health systems. Appropriate use of medicines contributes to the prevention, treatment and eradication of various diseases [1,2]. Access barriers to medicines limits the therapeutic capabilities of health personnel and jeopardizes patient health via reduced health service utilization as well as imposing financial burdens on users, which are often catastrophic [3].

Within resource constrained settings, low availability of medicines in health care units is particularly related to shortcomings in facility management, including through the purchasing, distribution or storage processes, as well as lack of training for personnel and insufficient investment in medicines in general [3]. Consequently, ensuring equitable and affordable access to medicines is an ongoing challenge for health systems.

In Mexico, the Seguro Popular (SP) has been implemented as a means to ensure timely and equitable access to medicines for poor populations [4–7]. SP is the main component of the Social Protection System in Health, which additionally includes the Protection Fund Against Catastrophic Medical Expenses and the Health Insurance Siglo XXI (previously known as, Insurance for a New Generation,). The SP was introduced in 2003 with the aim of ensuring the provision of health services and providing financial protection through a voluntary insurance to the populations that, due to their employment status (unemployed and self-employed), are excluded from social security [4–6].

The financial model input of the SP consists of federal government and state aid, with contributions from members according to their economic capacity [4]. Since its inception, the number of SP members has grown exponentially from approximately 2 million in 2003 to nearly 53 million by the end of 2012 [8,9]. In addition, funds allocated to SP have experienced a significant growth to 1,300%, from USD 382.6 million in 2004 to USD 5,087.6 million in 2012 (with an annual average/affiliated of USD 100) [10], of which up to 30% annually is intended for the purchase of medicines [7,9]. Currently, SP ensures access to a package of interventions called Universal Health Services Catalog (CAUSES), including 284 interventions and 522 medicines [10–13].

Despite SP’s growing financial protection, supply of medicines in Mexico’s public health units remains a barrier to accessing care [14,15]. Recent studies suggest that in 2008 the SP population had an 86.7% probability of incurring expenditure on medicines (among those who spent on health), with an average amount equivalent to 6.2% of disposable household income accounting for nearly 80% of household expenditure on health (6% higher than the national average) [16]. Results of external evaluations of SP 2006 show that demand for medicines has increased: 83.3% and 89.1% (in 2005 and 2006 respectively) of SP members who were treated as outpatients in Ministry of Health (MoH) units were prescribed medicines [17]. In 2002 the estimated figure for the same population was 55% [18].

Much of the published literature reports on the earlier years of SP implementation. Now that the government has declared success over meeting its goal of universal coverage, a study of SP affiliates access to prescribed medicines (ATPM) through primary care is warranted. This study aims to compare ATPM for SP and non-SP affiliated users of primary care services, taking into account the type of provider, socio-demographic profile health needs of users of these services, as well as the main barriers to ATPM for both insurance groups.

## Materials and Methods

### 2.1 Data, study population and key definitions

We performed a cross-sectional study with data collected by the National Survey of Health and Nutrition (ENSaNut) 2012. This survey used a stratified multistage sampling, probabilistic design and representation at national, state and stratified urban/rural levels in each of the 32 states of Mexico [19]. In particular, we analyzed the data from the primary care service user module and the associated households. The ENSaNut includes information from 194,758 individuals. The primary care service user module represents a random sample of 12,973 people who claimed having received primary care services during the two weeks prior to the survey and who received care from medical personnel. We obtained the data in an anonymized form for analysis from the public repository of the National Health and Nutrition Survey (ENSaNut). The local Committees of Research, Biosafety and Ethics at the National Institute of Public Health (INSP) in Mexico approved the survey. All subjects provide informed consent prior to taking part.

Access to prescribed medicines or ATPM was defined as receiving all medicines prescribed from primary care services, regardless of whether or not individuals had to pay. In principle, medicines could be obtained at the MoH facilities (the same place as the medical consultation) free of charge, or outside MoH facilities (e.g. private community pharmacies) where individuals have to pay for medicines. For both cases a dichotomous variable equal to one was used if the event occurred and zero was used if the event did not occur.

An individual who reported insurance affiliation to Seguro Popular at the time of the survey was classified as SP and those without any health insurance scheme were classified as non-SP (marked as 0).

The test sample included 6,123 individuals (around 4 million nationwide), with a sample loss of 13.6%. It is relevant to note that the analysis of key socio-demographic characteristics among both those included and those excluded from the study population suggests no statistical differences exist between the groups.

### 2.2 Analytic Strategy

The main socio-demographic characteristics of the study population according to SP membership are described. Through statistical independence tests (χ2 for categorical variables and t-student for continuous) [20,21] the main differences between the two groups of affiliation were described. Using the same tests, the ATPM and the main reasons for its non-occurrence were identified (lack of medicines in the care unit, remoteness, lack of time for the person who used health services were not included as part of the consultation received, lack of money, prices, or other). The influence of SP on ATPM was estimated in principle from the following specification:

ATPM=α+δSP+γX+ε (1)

Where X is a vector of control variables of such effect and ε the error term [21]. This model assumes that there are no "unobservable" omitted variables which influence both the decision to join SP and the probability of using health services. This assumption is feasible when the definition of ATPM is independent of the place where the prescribed medicines were obtained. Enrolling in SP is non-random and voluntary which means that the estimation of the SP’s influence on (δ) ATPM at the place of care (in the MoH), could be confounded by factors related to any differences associated with problems of self-selection [*corr*(*X*, *ε*) ≠ 0]. This could result in biased and inconsistent estimates of the influence of the SP [22–24]. The first step estimations used to resolve this issue followed as:

SP=ψZ+τ (2)

Where Z is a vector of instrumental variables, in such a way that such assumptions *corr*(*Z*, *SP*) ≠ 0 and *corr*(*Z*, *ε*) ≠ 0 are met [25].

The Z vector included the natural logarithm of the number of SP members at municipal-level between the years 2003–2010. This information is derived from administrative records of the National Commission for Social Protection in Health [26]. Previous studies have shown the validity of this instrumental variable [27,28]; it is highly correlated with the probability of being member of SP and not associated with the probability of receiving health care. However, unlike the aforementioned studies, our approach not only captures the penetration of the program at the municipal level and heterogeneity cross, it also incorporates the dynamics of expansion, anticipating that municipalities with greater penetration and higher expansion rates will include individuals with a higher probability of SP affiliation.

Additionally, X included the following control variables: reported health needs (acute, chronic or other) [29], the number of medicines prescribed (1, 2, ≥3), age, and sex. Other control variables at household level were sex, age, the head of houses’ education and employment status, indigenous status [30], social program beneficiary of Oportunidades (formerly Prospera), and total annual household expenditure per adult equivalent (in terciles) [31]. (3) The place of residence (city or town) was characterized by population size (rural: <2500 inhabitants, urban: 2500–100 thousand, metropolitan: >100 thousand) and degree of marginalization (very low/low, medium, high/very high) [32].

In order to estimate the association between SP and ATPM two approaches were proposed. First, considering the endogeneity problem mentioned, we adjust an IV model using 2-stage least squares (2SLS) [22]. I would suggest rephrasing as: Because both SP and ATPM are binary variables whose error terms might not be independent, a bivariate probit model or bi-probit was used to analyze the probability of ATPM. [22, 33,34]. Second, and following other studies [34], we contrasted our IV estimations using a naïve model that assumes exogeneity, and therefore directly adjusted (1). Both approaches include crude and adjusted estimations in order to control for any possible distortion of the association between SP and ATPM that would arise from differences in socio-demographic, health, and contextual characteristics included in the X vector. Marginal effects were also reported (in percentage points or pp) [22,35,36]. All analyses were performed using Stata 13.1 [37].

## Results

Table 1 shows the general characteristics of the study population (N = 4,064,495) according to SP membership. Some relevant differences include: (1) 20.6% of SP members (N = 2,753,713) and 15% of the non-SP population (N = 1,310,782) reported a chronic condition, while 69.9% and 77.5% of the respective groups reported having some acute condition; (2) 64.6% of SP members and 18.5% of non-members received health care in primary care MoH facilities, while 35.4% and 81.5% of the respective groups received care in a private clinic or pharmacy; (3) 55.7% of SP members and 60.3% of non-members were prescribed three or more medications; (4) the SP population lived in households whose head of house had an average of 1.7 years less schooling compared to non-SP affiliates. (5) Among the SP population, 9.5% belonged to indigenous households (3% more than the non-SP), 38.5% lived in households Oportunidades recipients (23.7% more than the non-SP) and 73.9% of households fall into terciles I and II for annual expenditure per equivalent adult (22% higher than non-SP). (6) With regard to the place of residence, 35% of the SP population lived in rural areas and 33.2% resided in environments of high/very high marginalization. For the non-SP population, these percentages were 16% and 15%, respectively.

**Table 1:** Differences in the study population according to Seguro Popular Affiliation.

	Seguro Popular	No Seguro Popular	Difference test p-value
n	4,558	1,565	
N	2,753,713	1,310,782	
%	67.7	32.3	
Individual Characteristics			
Health needs			
Acute	69.9 [67.9,71.9]	77.5 [74.4,80.3]	0.00
Chronic	20.6 [18.9,22.5]	15.0 [12.6,17.8]	
Other	9.43 [8.33,10.7]	7.52 [5.87,9.59]	
Place where care was received			
Ministry of Health (MoH)	64.6 [61.9,67.3]	18.5 [15.9,21.5]	0.00
Pharmacy units	18.6 [16.4,21.0]	35.5 [31.7,39.4]	
Private doctor	16.8 [15.2,18.6]	46.0 [41.9,50.2]	
Number of prescription medications			
1	11.4 [10.2,12.8]	11.2 [9.17,13.7]	0.06
2	32.9 [30.9,35.0]	28.5 [25.3,31.9]	
≥3	55.7 [53.4,57.9]	60.3 [56.6,63.9]	
Age Group			
0–4	22.2 [20.4,24.1]	25.1 [22.0,28.4]	0.00
5–19	29.9 [27.5,32.3]	27.0 [23.5,30.8]	
20–49	28.9 [27.0,30.9]	34.8 [31.7,38.0]	
50–69	15.1 [13.7,16.6]	10.4 [8.40,12.8]	
≥70	3.89 [3.20,4.73]	2.84 [1.93,4.17]	
Man	37.5 [35.6,39.4]	42.5 [38.8,46.3]	0.02
Home of residence			
Household head			
Man	78.0 [76.1,79.8]	76.5 [73.2,79.5]	0.39
Years old	44.7 [44.0,45.4]	45.0 [43.8,46.1]	0.63
Years of schooling	6.48 [6.29,6.67]	8.13 [7.69,8.57]	0.00
Work	76.3 [74.3,78.2]	76.9 [73.6,79.9]	0.75
Indigenous	9.54 [8.10,11.2]	6.63 [4.99,8.76]	0.02
Oportunidades beneficiary	38.5 [36.2,40.8]	14.8 [12.3,17.8]	0.00
Tercile of annual expenditure			
I	39.8 [37.4,42.1]	20.0 [17.1,23.3]	0.00
II	34.1 [32.1,36.2]	31.9 [28.0,36.1]	
III	26.1 [23.9,28.6]	48.1 [44.0,52.1]	
City or town			
Size			
Rural	35.0 [32.8,37.4]	15.9 [13.8,18.2]	0.00
Urban	24.6 [22.7,26.7]	15.9 [13.4,18.8]	
Metropolitan	40.4 [37.7,43.1]	68.2 [64.7,71.4]	
Degree of marginalization			
Very low/low	50.7 [47.7,53.6]	75.0 [71.7,78.1]	0.00
Medium	16.2 [13.9,18.7]	10.3 [8.04,13.0]	
High/very high	33.2 [30.3,36.2]	14.8 [12.5,17.4]	

Note: Estimates considering the survey design.

**Fig 1:**
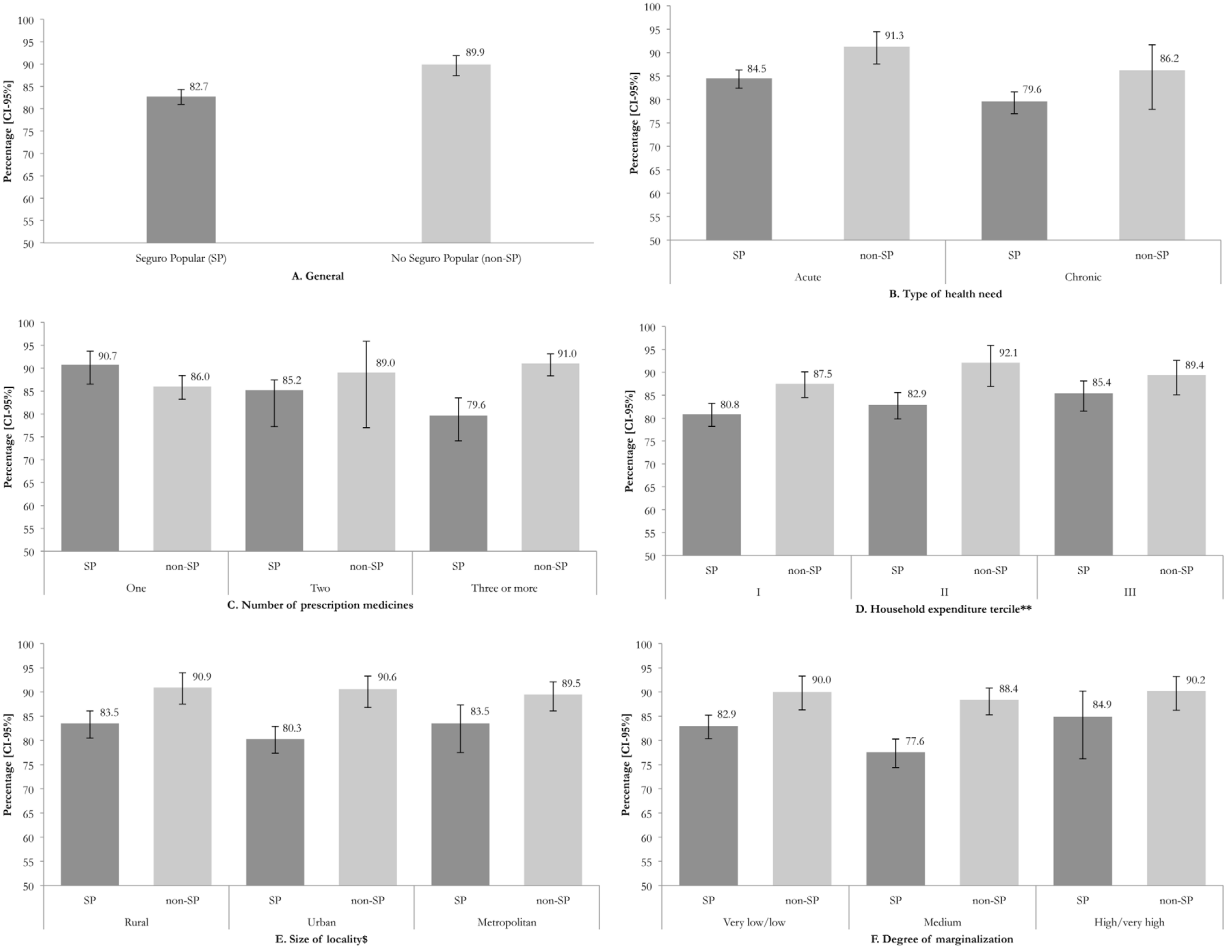
Access to prescribed medicines (ATPM) regardless of place according to individual characteristics and specific environment and affiliation to the Seguro Popular. Note: Estimates considering the survey design. *Estimated figures on the total population who received a prescription from medical personnel. **Calculation per adult equivalent. $ Rural: <2500 inhabitants, Urban: 2500–100000, Metropolitan: >100000.

At a national level, 82.7% of SP members and 89.9% of non-SP members reported ATPM in primary care services. The remaining individuals in both populations were unable to obtain either some or all of their prescribed medicines (Fig 1). ATPM changed according to individual and household characteristics as well as type of care received. The probability of ATPM decreased when there was an increase in the number of medicines, when there was lower household spending, and in the presence of chronic diseases. In terms of size of locality and degree of marginalization, no trend was detected.

**Fig 2:**
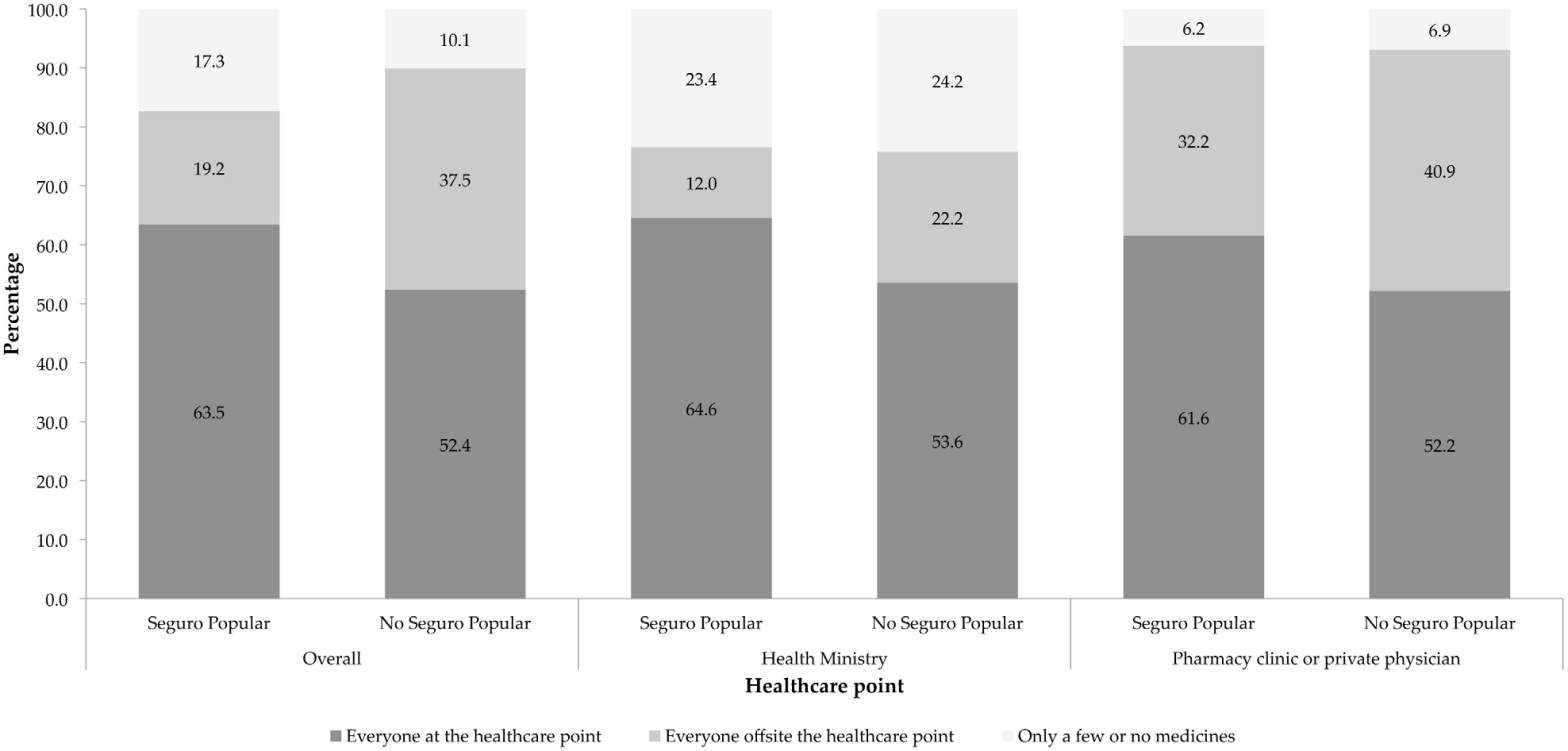
Access to prescribed medicines by type of care provider. Note: Estimates considering the survey design. *Indicator calculated on the total population who receive a prescription from medical personnel.

The descriptive analysis shows that, when stratified by type of institutions where health care was received, SP beneficiaries using the MoH reported greater ATPM than the non-SP members (64.6% v. 53.6%) (Fig 2). Interestingly, among beneficiaries of SP, the percentage of users who received either a portion or none of their prescription medicines was higher among those who used the MoH compared to those who used the private sector (23.4% v. 6.2%).

In nine out of ten cases, unavailability of medicines was reported as the reason for SP affiliated individuals experiencing no ATPM when receiving care at MoH institutions (Table 2). In contrast, when SP members reported no ATPM when receiving care in private institutions or pharmacy clinics, the primary reason was that the medicines seemed expensive or they lacked money to pay for the medicines (31%).

**Table 2:** Overall reasons for not obtaining medicines, according to the place where the health care was received and to affiliation to the Seguro Popular.

	Seguro Popular	No Seguro Popular	Difference test p-value
n	4,558	1,565	
N	2,753,713	1,310,782	
%	67.7	32.3	
PANEL A: General			
Lack of medicine	85.9 [82.4,88.8]	58.6 [44.7,71.2]	0.00
Did not know where to go, lack of time or cannot leave work	0.24 [0.06,0.95]	6.09 [1.56,20.9]	
They seemed expensive or lack of money	5.79 [4.12,8.07]	21.3 [12.2,34.4]	
Health care does not include medicines	4.18 [2.53,6.82]	7.45 [3.56,14.9]	
Other reason	3.90 [2.52,5.97]	6.63 [2.90,14.5]	
PANEL B: Users of health services of the Ministry of Health			
Lack of medicine	91.4 [88.2,93.8]	83.3 [67.8,92.2]	0.02
Did not know where to go, lack of time or cannot leave work	0.13 [0.12,0.15]	2.43 [0.66,8.49]	
They seemed expensive or lack of money	2.16 [1.26,3.66]	5.08 [2.85,8.90]	
Health care does not include medicines	2.18 [0.90,5.16]	0.38 [0.05,2.71]	
Other reason	4.12 [2.60,6.47]	8.80 [2.24,28.9]	
PANEL C: Users of pharmacy units or private clinics			
Lack of medicine	48.0 [37.0,59.1]	38.9 [24.2,55.9]	0.18
Did not know where to go, lack of time or cannot leave work	0.94 [0.13,6.61]	8.99 [1.74,35.5]	
They seemed expensive or lack of money	30.8 [21.6,41.9]	34.2 [18.2,54.7]	
Health care does not include medicines	17.9 [10.8,28.3]	13.1 [6.25,25.3]	
Other reason	2.36 [1.96,2.84]	4.91 [2.22,10.5]	

Note: Estimates considering the effect of the survey design.

Finally, Table 3 shows the influence of SP on the probability of ATPM. When only analyzing the group of individuals that obtained prescription medicines at the same place where they received health care, SP increases the probability of ATPM 10.3 percentage points compared to non-SP members (Model B.2). This association is reduced to 5.5 percentage points after adjusting for control variables. As in the previous model, the probability of ATPM is reduced among those who reported a chronic health need (v. acute condition), when the number of prescription medicines was ≥3 (v. having only one medicine prescribed), between users categorized in tercile III of annual spending (v. terciles I and III), as well as for residents of urban or metropolitan areas, and those living in low/very low marginalization areas.

**Table 3:** Comparison of access to prescribed medicines (*ATPM*) between those affiliated to Seguro Popular and those not affiliated.

	Probit Model		IV-Probit Model	
	Crude (B.1)	Adjusted (B.2)	Crude (B.3)	Adjusted (B.4)
	Marginal effects and CI95% reported			
Affiliation to the Seguro Popular	0.10**	0.06**	0.35**	0.29**
	[0.08,0.13]	[0.02,0.09]	[0.27,0.42]	[0.17,0.40]
Individual Characteristics				
Place of health care: Ministry of Health (Ref.: Private or pharmacy)		0.01		0.01
		[-0.02,0.04]		[-0.02,0.03]
Health need (Ref.: Acute)				
Chronic		-0.08**		-0.06**
		[-0.12,-0.04]		[-0.09,-0.04]
Other		-0.13**		-0.10**
		[-0.17,-0.08]		[-0.13,-0.06]
Number of prescribed medicines (Ref.: 1)				
2		-0.01		-0.01
		[-0.05,0.03]		[-0.04,0.03]
≥3		-0.06**		-0.05**
		[-0.10,-0.02]		[-0.08,-0.02]
Age		0.001**		0.001**
		[0.0004,0.002]		[0.0003,0.001]
Man		-0.01		-0.01
		[-0.04,0.01]		[-0.03,0.01]
Home of residence				
Household head				
Man		-0.04*		-0.03*
		[-0.07,-0.003]		[-0.05,-0.003]
Years old		-0.002**		-0.001**
		[-0.003,-0.001]		[-0.002,-0.001]
Years of schooling		-0.005**		-0.004**
		[-0.009,-0.002]		[-0.01,-0.002]
Work		0.01		0.01
		[-0.02,0.05]		[-0.02,0.04]
Indigenous		-0.004		-0.01
		[-0.05,0.04]		[-0.04,0.03]
Oportunidades beneficiary		0.01		0.01
		[-0.02,0.04]		[-0.01,0.03]
Tercile of annual expenditure (Ref.: III)				
I		0.09**		0.07**
		[0.06,0.12]		[0.04,0.09]
II		0.06**		0.05**
		[0.03,0.09]		[0.02,0.07]
City or town				
Size (Ref.: Rural)				
Urban		-0.09**		-0.07**
		[-0.13,-0.06]		[-0.10,-0.05]
Metropolitan		-0.06**		-0.03+
		[-0.10,-0.02]		[-0.06,0.002]
Degree of marginalization (Ref.: Very low/low)				
Medium		-0.008		-0.01
		[-0.05,0.03]		[-0.04,0.02]
High/very high		0.04*		0.03*
		[0.01,0.08]		[0.003,0.06]
Observations	6,123	6,123	6,123	6,123
Log likelihood	-3,999	-3,887	-7,347	-7,245
LR χ2	52.76	276.7	----	----
Prob > χ^2^	0.000	0.000	----	----
Wald χ2	----	----	430.5	653.1
Prob > χ^2^	----	----	0.000	0.000
AIC	8,002	7,816	14,719	14,551
rho (ρ)	----	----	-0.556	-0.477
Likelihood-ratio test of rho (ρ) = 0				
χ^2^	----	----	30.70	12.48
Prob > χ^2^	----	----	0.000	0.000

Note:

**P <0.01

*p <0.05

^+^p <0.1

## Discussion

Access to prescribed medicines or ATPM is one of the most sensitive indicators of the performance of the Mexican health system. This study contributes to the empirical analysis of a major health programs that the Mexican government has implemented in the last decade; the Seguro Popular or SP [7]. The main contribution of this study arises from the result comparing ATPM prescribed in ambulatory care between members of SP and those not affiliated with SP. Barriers to ATPM for the SP affiliated population result in overall lower access to medicines compared to that in the non-SP member population, one-fifth (17%) of SP beneficiaries did not obtain some or all of their prescribed medicines. This percentage is higher than was estimated in the non-SP population (10% of non-SP users failed to receive all of their prescription medicines).

This analysis also revealed that regardless of whether ambulatory health care is provided, being affiliated with SP has no influence on ATPM. Some explanatory factors include that one-third of SP affiliates seek primary care outside MoH facilities, which would result in out-of-pocket expenses to obtain medicines. In this case ATPM depends on one’s ability to pay rather than their affiliation (74% of the SP are terciles I and II v. 52% of the non-SP group).

This study suggests that care in MoH facilities is a key factor in analyzing the influence of SP on ATPM. This is not surprising considering that medicines are only provided free to SP beneficiaries at the MoH point of care. In crude terms, the percentage of ATPM for SP is 64.6% compared to the non-SP group at 53.6%. Although the analytic model shows a high probability (28.5 percentage points) for SP affiliates receiving ATPM in MoH facilities compared to non-SP individuals, it is concerning that ten years after the SP’s implementation 35.4% of SP affiliates still have to obtain medicines outside of MoH facilities.

Given the policy goals and implementation of a program like SP, ATPM is not yet at the level expected. SP guarantees ATPM for those medicines included in the formulary free of charge at the point of care. CAUSES, the SP insurance formulary, states that 285 interventions are included in the coverage, which represents 100% coverage for primary care visits and up to 90% coverage of secondary care consultations (SSA, 2014). International recommendations suggest 80% or greater availability to essential generic medicines in both public and private institutions [38].

Finally, the findings of this study demonstrate that users seeking care for chronic health problems as opposed to an acute and users prescribed ≥3 medications have a lower probability of obtaining their prescribed medicines. ATPM for chronic health needs is lower than for acute problems; this finding is supported by previous research [39]. Users with chronic conditions have a higher probability of comorbidities than those without resulting in higher out-of-pocket expenditure and the need for financial protection. The ENSANUT 2012 reveals that 47% of patients diagnosed with diabetes mellitus also have a diagnosis of high blood pressure [40].

Some limitations should be noted. First, the type of information source used (a population survey) has a transversal character, meaning it is subject to self-report biases as well as limitations in the ability to establish causal inference. Secondly, the study was unable to estimate the influence of SP solely among users of MoH. This limitation arises due to the small number of MoH service users that are not affiliated with SP. For every four users affiliated with SP, there is one non-SP user. Finally, we limited our study to access prescribed medicines rather than medicines in general, as the need for receiving pharmacotherapy is established by a health care provider. Despite these limitations, we believe that the following constituents promote assurance of the results:

1. The benefits of the sampling design of the survey used (probabilistic, nationally representative, state and urban/rural strata, and oversampling of households in greatest need of the country).
2. The use of instrumental variables to adequately correct the endogeneity problem for both discrete and unobservable variables (compared to other methods such as matching by propensity score), as well as the use in the analysis of control variables widely suggested in the international literature as determinants of service use. In order to test the validity of our results we used propensity score matching. The results produced are very similar to the ones reported in our manuscript (data not shown).

In conclusion the results of this study point to pending tasks that must be addressed before universal health coverage is to be achieved, specifically. Among the affiliated with SP, the lack of ATPM results in out-of-pocket spending on medicines and financial vulnerability of Mexico’s poor population. Apart from ATPM, the study results highlight the fact that improving ATPM in MoH facilities alone will only partly increase overall access to medicines among lower-income households. Results show that one-third of SP affiliates decided to seek care outside MoH facilities. If the government aims to guarantee ATPM via universal coverage (SP), user preferences must be considered. As such, it is necessary to identify factors discouraging the demand for MoH services, thus ensuring ATPM based on the health needs of disadvantaged populations in Mexico.

## References

[1] Organización Mundial de la Salud (OMS). Medicamentos esenciales. Disponible en: http://www.who.int/topics/essential_medicines/es/. (Last accessed: 5 May 2013).

[2] Organización Mundial de la Salud (OMS). Medicamentos esenciales. 10 datos sobre los medicamentos esenciales. Disponible en: http://www.who.int/features/factfiles/essential_medicines/es/index.html. (Last accessed: 5 May 2013).

[3] Garrido-Latorre F, Hernández-Llamas H, Gómez-Dantés O. 2008 Surtimiento de recetas a los afiliados al Seguro Popular de Salud de México. *Salud Pública Mex* 50 supl 4:S429–S436. 10.1590/s0036-36342008001000003 19082253

[4] Secretaría de Salud (SeSa). 2006 Utilización de servicios y trato recibido por los afiliados al Seguro Popular de Salud In: Secretaría de Salud. Sistema de Protección Social en Salud. Evaluación de procesos. Mexico City: Secretaría de Salud, 2006: 39–57.

[5] Gómez Dantés O, Ortiz M. 2004 Seguro popular de salud: Siete perspectivas. Salud Pública De México, 46(6), 585–588. 15624862 10.1590/s0036-36342004000600013

[6] King G, Gakidou E, Imai K, Lakin J, Moore R, Nall C, et al 2009 Public policy for the poor? A randomized assessment of the mexican universal health insurance programme. The Lancet, 373(9673), 1447–1454. 10.1016/S0140-6736(09)60239-7 19359034

[7] Knaul FM., González-Pier E, Gómez-Dantés O, García-Junco D, Arreola-Ornelas H, Barraza-Lloréns M, et al 2012 The quest for universal health coverage: Achieving social protection for all in Mexico. The Lancet, 380(9849), 1259–1279. 10.1016/S0140-6736(12)61068-X 22901864

[8] Comisión Nacional de Protección Social en Salud. 2013. Cifras del Seguro Popular: Afiliación histórica. Disponible en: http://www.seguro-popular.salud.gob.mx/index.php?option=com_content&view=article&id=552&Itemid=481. (Last accessed: 13 July 2013).

[9] Comisión Nacional de Protección Social en Salud. 2012. Libro Blanco. Sistema de Protección Social en Salud. “Seguro Popular”. Disponible en: http://www.seguro-popular.salud.gob.mx/images/contenidos/Transparencia/rend_ctas_libros_bcos/seguropopular.pdf. (Acceded 2013 June 19).

[10] Sistema de Protección Social en Salud. Informe de resultados 2012. Disponible en: http://gaceta.diputados.gob.mx/Gaceta/62/2013/feb/InfSPSS-20130201.pdf. (Acceded 2013 June 19).

[11] Sistema de Protección Social en Salud. 2012. Informe de resultados del SPSS Enero-Diciembre 2012. Secretaría de Salud. Disponible en http://www.seguro-popular.salud.gob.mx/index.php?option=com_content&view=article&id=277&Itemid=293. (Acceded 2013 June 17).

[12] Sistema de Protección Social en Salud. Informe de resultados 2011. Disponible en: http://www.slpsalud.gob.mx/archivos/Seguro-Popular/PEF/2011/Informe_Resultados_2011.pdf. Consultado: 19 de junio de 2013.

[13] Instituto Nacional de Salud Pública. 2012. Evaluación Externa del Sistema de Protección Social en Salud 2012. Disponible en: http://salud_2013.salud.gob.mx/codigos/columnas/evaluacion_programas/pdf/EXT12_SPSS_SE.pdf. (Acceded 2013 June 24).

[14] Wirtz VJ, Russo G, Kageyama ML. 2010 Acceso a medicamentos para los usuarios del primer nivel de los servicios de salud: análisis de las encuestas nacionales de salud 1994, 2000, 2006. Salud Pública Mex; 52:32–40.

[15] López Moreno S, Granados Cosme JA, coordinadores. El abasto de medicamentos en México. México, DF: Universidad Autónoma Metropolitana, Unidad Xochimilco, 2010.

[16] Wirtz VJ, Santa-Ana-Téllez Y, Servan-Mori E, Avila-Burgos Leticia. 2012 Heterogeneous Effects of Health Insurance on Out-of-Pocket Expenditure on Medicines in Mexico. Value Health 15: 593–603. 10.1016/j.jval.2012.01.006 22867767

[17] Secretaría de Salud de México (SeSa). 2006. Sistema de Protección Social en Salud: Evaluación de Procesos. Primera edición. México 2007. Available in http://salud_2013.salud.gob.mx/codigos/columnas/evaluacion_programas/cnpss1.html.

[18] Secretaría de Salud (SeSa)-Dirección General de Evaluación del Desempeño (DGED). 2006. Síntesis Ejecutiva 15. Surtimiento de recetas en los Servicios Estatales de Salud y en los Servicios de Salud del DF. Available in http://www.salud.gob.mx/unidades/evaluacion/publicaciones/publicaciones.htm

[19] Romero-Martínez M, Shamah-Levy T, Franco-Núñez A, Villalpando S, Cuevas-Nasu L, Gutiérrez JP, et al 2013 Encuesta Nacional de Salud y Nutrición 2012: diseño y cobertura. Salud Pública Mex; 55 supl 2:S332–S340. 24626712

[20] Greenwood P.E., Nikulin M.S. 1996 A guide to chi-squared testing. Wiley, New York. ISBN 0-471-55779-X.

[21] McDonald J.H., Handbook of Biological Statistics Sparky House Publishing, Baltimore, 2008.

[22] Wooldridge JM. 2002 Econometric analysis of cross section and panel data. MIT Press, Cambridge, 2002.

[23] Angrist J, Krueger A. 2001 Instrumental Variables and the Search for Identification: From Supply and Demand to Natural Experiments. Journal of Economic Perspectives 15(4):69–85.

[24] Angrist J, Imbens GW, Rubin D. 1996 Identification of Causal Effects Using Instrumental Variables. Journal of the American Statistical Association 91 (434): 444–455.

[25] Heckman JJ. 1997 Instrumental variables: a study of implicit behavioral assumptions used in making program evaluations. J. Hum. Resour 32: 441–462.

[26] Comisión Nacional de Protección Social en Salud. 2010 Datos administrativos de la afiliación al Seguro Popular a nivel de las localidades y fecha de incorporación de las municipalidades. México, DF: Secretaría de Salud, 2010.

[27] Galárraga O, Sosa-Rubí S, Salinas-Rodríguez A, Sesma-Vázquez S. 2010 Health insurance for the poor: impact on catastrophic and out-of-pocket health expenditures in Mexico. Eur J Health Econ 11 (5): 437–447. 10.1007/s10198-009-0180-3 19756796 PMC2888946

[28] Sosa-Rubí SG, Galárraga O, Harris JE. 2009 Heterogeneous impact of the “Seguro popular” program on the utilization of obstetrical services in Mexico, 2001–2006: A multinomial probit model with a discrete endogenous variable. Journal of Health Economics 28(1): 20–34. 10.1016/j.jhealeco.2008.08.002 18824268 PMC2790917

[29] Wirtz VJ, Serván-Mori E, Heredia-Pi I, Dreser A, Ávila-Burgos L. 2013 Utilización y gasto en medicamentos en México: avances y retos en el acceso equitativo a medicamentos. Salud Pública Mex 55 supl 2:S112–S122. 24626686

[30] Comisión Nacional para el Desarrollo de los Pueblos Indígenas (CDI). 2009. Los hogares y la población indígena. Available in: http://www.cdi.gob.mx/index.php?id=211&option=com_content&task=view, 2009. (Last accessed: 1 October 2012).

[31] Teruel GL, Rubalcava L. 2005. Escalas de Equivalencia para México, D.F. Serie: Documentos de Investigación. SEDESOL. Documentos de investigación 23, SEDESOL, 2005.

[32] Consejo Nacional de Población (CONAPO). 2010. Índice de marginación por localidad. Available in http://www.conapo.gob.mx/es/CONAPO /Indice_de_Marginacion_por_Localidad_2010. (Acceded 2012 November 11].

[33] Verbeek M.J.C.M. (2012). A Guide to Modern Econometrics, 4th edition Chichester: John Wiley and Sons.

[34] Galárraga O, Sosa-Rubí S, Salinas-Rodríguez A, Sesma-Vázquez S. 2010 Health insurance for the poor: impact on catastrophic and out-of-pocket health expenditures in Mexico. Eur J Health Econ 11 (5): 437–447. 10.1007/s10198-009-0180-3 19756796 PMC2888946

[35] Cameron AC and Trivedi PK. 2009. Microeconometrics Using Stata. College Station, TX: Stata Press, 2009.

[36] Greene WH. 2003. Econometric analysis. Prentice Hall, Upper Saddle River, 2003.

[37] Stata Corp. LP. Stata/SE 12.1 for Windows XP 64 bits. College Station Texas, USA: Stata Corp LP, 2011.

[38] World Health Organization (WHO). 2012. A draft comprehensive global monitoring framework, including indicators, and a set of voluntary global targets for the prevention and control of non-communicable diseases. A/NCD/INF./1 31 October 2012. Ginebra, OMS, 2012.

[39] Cameron A, Roubos I, Ewen M, Mantel-Teeuwisse AK, Leufkens HG, Laing RO. 2011 Differences in the availability of medicines for chronic and acute conditions in the public and private sectors of developing countries. Bull World Health Organ 1;89(6):412–21. 10.2471/BLT.10.084327 21673857 PMC3099556

[40] Hernández Ávila M, Gutiérrez JP. Documento Analítico de la ENSANUT 2012. Diabetes mellitus: la urgencia de reforzar la respuesta en políticas públicas para su prevención y control. Available in: http://ensanut.insp.mx/doctos/analiticos/DiabetesMellitus.pdf (Acceded 2013 August 20).

